# Determination of Bioavailable Aluminum in Natural Waters in the Presence of Suspended Solids

**DOI:** 10.1002/etc.4448

**Published:** 2019-07-25

**Authors:** Patricio H. Rodriguez, Jose J. Arbildua, German Villavicencio, Paola Urrestarazu, Margaret Opazo, Allison S. Cardwell, William Stubblefield, Eirik Nordheim, William Adams

**Affiliations:** ^1^ PHR Consulting Santiago Chile; ^2^ Chilean Mining and Metallurgy Research Center Santiago Chile; ^3^ Oregon State University, Corvallis Oregon USA; ^4^ European Aluminium Association Brussels Belgium; ^5^ Red Cap Consulting, Lake Point Utah USA

**Keywords:** Water quality criteria, Metal toxicity, Bioavailability, Aluminum method, Aluminum extraction

## Abstract

Analyses of natural waters frequently show elevated levels of total aluminum (Al) attributable to acid extraction of Al from the total suspended solids (TSS) minerals. Hence, there is a need for an analytical method that measures only bioavailable Al. Natural waters high in TSS were collected to study the chronic effects of Al on *Ceriodaphnia dubia*. In the collected waters TSS ranged from 30 to 411 mg/L; total Al concentrations ranged from 2.0 to 44.8 mg/L. The TSS in natural waters inhibited reproduction of *C. dubia* up to 40% in comparison to the same filtered waters. This inhibition did not correlate with the concentration of TSS or total Al; it was attributed to nutritional deficiency and was prevented by increasing the food supply. To demonstrate that toxicity can be measured in natural waters, samples with elevated TSS were spiked with soluble Al, and survival and reproduction were measured in chronic studies performed at pH 6.3 and 8.0. To properly characterize the Al concentrations in the toxicity studies, a method was needed that could discriminate bioavailable Al from mineral forms of Al. An extraction method at pH 4 for bioavailable Al was developed and evaluated using *C. dubia* chronic toxicity studies in the presence of TSS. It is concluded that the proposed method is better able to discriminate chronic toxicity effects attributable to bioavailable Al from mineralized nontoxic forms of Al compared with existing methods using total or total recoverable Al (i.e., extraction at pH ≤ 1.5). We propose that this new method be used when assessing the potential for Al in natural surface waters to cause toxicity. *Environ Toxicol Chem* 2019;38:1668–1681. © 2019 The Authors. *Environmental Toxicology and Chemistry* published by Wiley Periodicals, Inc. on behalf of SETAC.

## INTRODUCTION

Recent research investigating the relationship between water chemistry and aluminum (Al) toxicity to aquatic organisms has been published (Adams et al. 2018; Cardwell et al. 2018; DeForest et al. 2018; Gensemer et al. 2018; Santore et al. 2018; Wang et al. 2018). Toxicity was shown to vary as a function of pH, dissolved organic carbon (DOC), and hardness, with biological responses correlating with total Al. No direct relationship between dissolved Al and toxicity was shown for a wide variety of aquatic organisms in chronic toxicity studies.

The publications mentioned in the previous paragraph have clearly demonstrated that, although toxicity of Al can be explained by the dissolved Al^3+^ at pH 5.0 or lower, the toxicity of Al in circumneutral waters does not correlate with dissolved Al. This is in contrast to the transition metals (e.g., cadmium, copper, lead, nickel, zinc). For these metals the US Environmental Protection Agency (USEPA) has based its criteria on the dissolved fraction (US Environmental Protection Agency 1993).

Toxicity of Al appears to be attributable to both ionoregulatory effects at acidic pH values and physical effects at neutral or alkaline pH conditions. Physical effects are often reported as asphyxiation attributable to coating of the respiratory membranes with Al hydroxide precipitates (Witters et al. 1996; Gensemer and Playle 1999; Teien et al. 2006). It is important to note that most surface waters are circumneutral (pH 6–8.5), where Al occurs as Al hydroxide species or mineral‐phase particulates, both of which are removed by filtration. These hydroxide precipitates can be seen visually as the solubility limit of Al is exceeded when the pH increases above 6.0. The Al precipitates formed at circumneutral to alkaline conditions can be removed by filtration, thereby eliminating toxicity in laboratory tests.

Assessing the toxicity of Al under circumneutral pH conditions thus poses a unique challenge because monomeric species of Al quickly transform into insoluble polymers which precipitate out of solution. During this transformation period, the formation of short‐lived or transient forms of polymeric Al, such as colloidal and amorphous forms, can exist on the scale of a few minutes to hours, whereas more stable crystalline forms (larger Al polymers and minerals) can take days to fully form (Sposito 1996). Transient species responsible for toxicity of Al are, therefore, unlikely to exist long enough to be a chronic risk to aquatic organisms except in mixing zones, where Al‐rich acidic waters meet more neutral waters, leading to precipitation of Al (Kroglund et al. 2001).

The solubility of Al is highly dependent on pH and, to a lesser extent, DOC and temperature. The solubility limit of Al can be as little as 20 μg/L at pH 6.5 and > 1000 μg/L at pH 5 (Santore et al. 2018). Because of the relationship between pH and Al solubility, acidification of surface waters can result in mobilization of the metal from solid‐phase materials (minerals, soils, suspended particles); or conversely, if the pH of a low‐pH, Al‐rich water is increased, precipitation of Al can result. The complexity of Al speciation as a function of pH raises questions regarding how to measure Al in circumneutral waters such that bioavailable forms are discriminated from other Al‐containing phases such as suspended solids rich in Al.

Regulatory bodies frequently recommend measurement of total recoverable Al (US Environmental Protection Agency 1994a) or acid‐soluble Al (US Environmental Protection Agency 1988). However, surface waters typically contain naturally occurring suspended solids that contain Al in the forms of oxides or silicates. Analytical determinations using strong acid result in most or all of the inert nontoxic Al present in solid particles being dissolved and the metal being reported as “total or total recoverable” Al. In surface waters with elevated suspended solids, the Al contributed from the suspended solids can result in Al concentrations that significantly exceed the previous USEPA chronic water quality criterion of 87 µg/L when measured as acid‐soluble or total recoverable (US Environmental Protection Agency 1988) and thus being listed on the USEPA 303d list as impaired waters (US Congress 1972). However, it is doubtful whether these total Al concentrations are bioavailable and, therefore, contribute to toxicity.

The present study was undertaken to develop a method that could appropriately measure Al not associated with total suspended solids (TSS) in surface waters for regulatory purposes and to demonstrate that the method is capable of measuring the fraction of Al responsible for toxicity. We present data in support of this new method and a demonstration that the toxicity of Al to *Ceriodaphnia dubia* can be assessed in waters high in suspended solids and the toxicity attributed to the Al which was added in a bioavailable form and not the suspended solids or the Al in the suspended solids. *Ceriodaphnia dubia* was selected because it is known to be sensitive to Al (Cardwell et al. 2018; Gensemer et al. 2018). Two rivers in Chile (Colorado and Tinguiririca), known to be high in suspended solids with little or no anthropogenic input, were selected as the initial test waters. As a further assessment of the new analytical method, water samples were subsequently collected from several large rivers in the United States, some of which are listed as impaired for Al, and analyzed for total, acid‐soluble, dissolved, and pH 4–extracted Al. These rivers represent a wide range of pH, DOC, hardness, and TSS levels and, as such, are representative of different water conditions in many parts of the world.

## METHODS

### Test substances

The test substances used in the execution of the experiments were Al nitrate nonahydrate (Chemical Abstracts Service [CAS] no. 7784‐27‐2, extra pure; Merck), which was used to conduct toxicity tests; Al oxide (Al_2_O_3_; SO 143; aluminium oxid stade (AOS)); sodium aluminate anhydrous (European Inventory of Existing Commercial Chemical Substances no. 235‐487‐0; Riedel‐de Haën); and atomized Al granules (Al 99.5%, <10 µm, MEP 105 RE0902; ECKA Granules). These substances were used in toxicity studies to demonstrate that they are nontoxic and not able to release Al by means of a pH 4 extraction. Kaolin (China Clay) powder (CAS no. 1332‐58‐7, lot no. 817820; Aqua Solutions) was used in some toxicity studies to mimic suspended solids in natural water samples.

### Test organism

The test organism used in the studies was the cladoceran *C. dubia*. An in‐house culture (batch no. CD030715) was originally obtained from MicroBioTests.

### Natural and artificial test media

One type of synthetic medium (test water) used in the experiments was a moderately hard water (USEPA 84 medium) composed of 60 mg/L CaSO_4_ × 2H_2_O, 60 mg/L MgSO_4_, 96 mg/L NaHCO_3_, and 4 mg/L KCl. An additional assay medium, simulating the Colorado River water, was prepared according to the following composition: 484 mg/L CaSO_4_ × 2H_2_O, 72 mg/L MgSO_4_, 266 mg/L NaHCO_3_, and 4 mg/L KCl (all the salts are Merck Pro analysis grade). After preparation, both media had an initial pH of approximately 8, which was ideal for studies at pH 8.0. To obtain pH 6.3, both media were titrated to pH 6.3 in the presence of 5 mM of buffer 2‐morpholinoethanesulfonic acid (MES).

When different medium conditions were used, such as with different pH values, natural or synthetic (kaolin) TSS content, soluble Al or other sources of Al, the medium was prepared and equilibrated for 3 h before the testing was started. This is consistent with the Al toxicity studies reported by Cardwell et al. (2018) and Gensemer et al. (2018).

Natural waters high in TSS were collected from the Colorado River (33°30′36.6′′S and 70°12′17.7′′W) in the Santiago Metropolitan Region of Chile and the Tinguiririca River at La Rufina site (34°44′23.2′′S and 70°45′10.7′′W) in the O'Higgins Region, Chile. Waters were collected in this region as the method development research was performed in Santiago, Chile. To avoid any anthropogenic contribution in the watershed, the rivers were sampled high in the Andes mountains, against the flow of the rivers from the stream surface (1 m deep), in new 1% HCl–washed, 5‐L, high‐density polyethylene plastic bottles.

### Toxicity testing methodology

*Ceriodaphnia dubia* were cultured as prescribed in USEPA methodology (US Environmental Protection Agency 2002). Neonates used for the toxicity tests were obtained from individual cultured organisms and were less than 24 h old at test initiation. This organism was selected because of its sensitivity to Al and because it is widely used in the development of water quality criteria. *Ceriodaphnia dubia* offers the advantage of being usable in a chronic bioassay in only 7 d. This was important because of the number of tests that were needed.

*Ceriodaphnia dubia* neonates were exposed to 5 test concentrations plus a control. Ten replicates per concentration, containing one neonate/test vessel, were exposed for 7 d (3 broods). Tests were performed in 30‐mL precleaned, disposable polypropylene cups containing 25 mL of test solution. Animals were fed daily with 0.1 mL of yeast, trout chow, and cerophyll and 0.1 mL algal suspension of *Raphidocelis subcapitata* (formerly known as *Pseudokirchneriella subcapitata*). The organisms were transferred to the fresh test solutions daily.

The animals were incubated in a growth chamber (Forma Scientific; model 3744) with a 16:8‐h light:dark cycle under normal fluorescent illumination with a light intensity of 10 to 20 µE/m^2^/s. The temperature was maintained at 25 ± 1 °C. Adult survival and fecundity (number of neonates per female) were assessed daily. Ambient temperature was recorded during the test with a maximum to minimum VWR brand thermometer. Daily pH was measured potentiometrically (USEPA method 150.1, pH‐meter; Beckman; model 340), and conductivity was measured with a conductivity meter (Mettler Toledo; model MC126‐2M).

Measurements of Al (total, dissolved, and pH 4–extracted) were made at time 0 h (following a 3‐h aging period) before the organisms were added to the test solutions. A 10‐mL aliquot was taken from each test concentration and analyzed as described in the *Chemical measurements* section.

### Summary of tests performed

The following toxicity studies were performed as part of this method‐development research program.

1. *Effect of TSS on C*. dubia *survival and reproduction*. To evaluate the toxicity of Al to *C. dubia* in the presence of TSS, it was necessary to first demonstrate that the daphnids could survive and reproduce in natural waters high in suspended solids. This is not a normal environment for this species because they are most often found in lakes and calm streams low in TSS. Water samples from the Colorado and Tinguiririca Rivers in Chile were collected, and a complete chemical characterization of the water was performed (Supplemental Data, Tables S1 and S2). The effect of TSS on *C. dubia* survival and reproduction in Colorado River water with increasing concentrations of TSS (2–255 mg/L) was characterized at pH 6.3 and 8. Tests were also performed using 1 and 2 times the normal amount of food in the presence of TSS to evaluate reproductive success.
2. *Toxicity of Al in waters naturally high in TSS*. A key question was whether it is possible to measure the intrinsic toxicity of Al in natural waters with high TSS which is not by itself toxic when sufficient food supplies are available. To evaluate this scenario, Colorado River water (pH 8.0) with elevated TSS (61 mg/L and total Al 3.8 mg/L) was spiked with increasing concentrations of soluble/bioavailable Al, in the form of Al(NO_3_)_3_ × 9H_2_O, and 7‐d chronic toxicity tests were performed with *C. dubia*.
3. *Ability of acid extraction methodology to differentiate bioavailable Al in natural waters from mineralized particulate forms of Al*. To specifically assess the intrinsic effect of Al exposures to aquatic organisms, it is necessary to distinguish between bioavailable Al and mineralized forms of the Al metal present in suspended solids (typically as Al silicates). To evaluate the importance of the strength of the acid digestion on recovery of Al, an Al acid extraction screening test was performed in water from the Colorado River (natural TSS) and in USEPA 84 medium in the presence of artificial TSS (kaolin clay). Water samples were adjusted to different pH values and extracted after 1 h of shaking on an orbital shaker at 100 rpm. These 2 waters contained TSS concentrations designed to result in equal amounts of total Al (44.8 mg/L).
4. *Dissolution kinetics—pH 4 extraction versus USEPA pH 1.5 procedure*. The Al dissolution kinetics in USEPA medium, with and without TSS (50 mg/L), were measured over time using the pH 4 method and compared with total Al measurements. The test medium had a pH of 6.3, and 1 mg/L of soluble Al was added. The TSS present in the tests, when totally dissolved, contained 5 mg/L mineralized Al.
5. *Dose–response relationship between toxicity and pH 4 acid–extractable Al*. Several chronic bioassays were performed with *C. dubia* to demonstrate a dose–response relationship between pH 4–extracted Al concentrations and inhibition of reproduction. The experimental design consisted of different test waters with and without TSS and spiked with bioavailable Al as follows: 1) a test water simulating the water quality characteristics of the Colorado River without TSS, 2) natural Colorado River water with no TSS, 3) natural Colorado River water with 61 mg/L TSS containing 4 mg/L of mineralized Al, 4) simulated Colorado River water with 200 mg/L of TSS, and 5) simulated Colorado River water (in terms of hardness) with 104 mg/L of kaolin clay (containing 22 mg/L of mineralized Al) at pH 6.3. To obtain TSS for test 4, natural water samples containing TSS were filtered to obtain the solid fraction, and then the filtered material was resuspended in the appropriate volume of test water to reach 200 mg/L containing 22 mg/L of total mineralized Al.
6. *Toxicity assessment using the pH 4 acid extraction method with several Al compounds*. To assess the relationship between toxicity and extracted Al using the pH 4 method, several Al compounds with a range of solubilities were independently assayed using *C. dubia* in Colorado River water with 30 mg/L TSS that contributed 2 mg/L mineralized Al when measured as total Al. The added Al concentrations of (2.7–4.0 mg/L) were selected to observe a reduction in reproduction of approximately 50% (50% effect concentration [EC50]). The tests contained 2.7 to 4 mg/L added Al and 2 mg/L Al derived from the TSS for a total of 4.7 to 6 mg/L when assayed as total Al.

### Chemical measurements

Water samples from toxicity tests and from natural surface waters were collected for the following analyses: total Al, acid‐soluble Al, pH 4–extracted Al, and dissolved Al. A short description of each term is provided in Table 1. The degree of aggressive Al extraction provided by the method from strongest to weakest is total Al > acid‐soluble Al > pH 4–extracted Al > dissolved Al. The method and terminology utilized for each analysis are vital to the interpretation of the concentration and type (i.e., mineral phase, dissolved) of measured Al.

**Table 1:** Methods used for Al analysis

Terminology	Short method description
Total Al^a^	Different methods for "total" and "total recoverable" exist, which entail different methods of acid digestion. For our Al studies, "total" has been processed as ufiltered sample, acidified with HNO_3_ to pH 1–2, then analysis (ICP‐OES; US Environmental Protection Agency 1994b).
Acid‐soluble Al (USEPA 1988)	Acidify unfiltered sample with 1 + 1 nitric solution to a pH of 1.65–1.85, digest for at least 16 h, filter through 0.45‐µm filter. Acidified with HNO_3_. Analysis by ICP‐OES (US Environmental Protection Agency 1994b).
pH 4 extracted Al	Add sodium acetate buffer solution to sample to a pH of 4.0–4.2. Agitate for 3 h (per P. Rodriguez, personal communication: changed to 3 h from original 1 h) at 100 rpm. Then filter through 0.45‐µm filter. Acidified with HNO_3_ to pH 1–2. Analysis by ICP‐OES (US Environmental Protection Agency 1994b).
Dissolved Al (0.45 µm filtration)	Operationally defined as a sample filtered through 0.45‐µm filter, then preserve with HNO_3_ to pH 1–2, then analysis (ICP‐OES) (US Environmental Protection Agency 1994b).

^a^ For the present study, we did not utilize the method referred to as “total recoverable” Al. This method is the most aggressive and utilizes strong acid (pH < 1) and heat to digest all solids. This method is frequently used for regulatory purposes. When analyzing waters very low in total suspended solids, the results obtained by total recoverable Al, total Al, and acid‐soluble Al are all equivalent.

Because of the high TSS content of the natural Chilean waters, samples were filtered through 0.45‐µm membranes before hardness and alkalinity determinations. Hardness was determined by titration with ethylenediaminetetraacetic acid (US Environmental Protection Agency 1982, method 130.2). Alkalinity was determined by potentiometric titration (US Environmental Protection Agency 1974, method 310.1).

Dissolved organic carbon was determined by catalytic combustion of the samples (US Environmental Protection Agency 1974, method 415.1) with a Tekmar Dohrmann model Apollo 9000 carbon analyzer.

Concentrations (dissolved) of Na^+^, K^+^, Ca^2+^, and Mg^2+^ ions in natural waters were determined by atomic absorption spectrophotometry (US Environmental Protection Agency 1990, method 7000 A) using a Perkin Elmer model PinAAcle 900 instrument. Chloride and sulfate were determined by ion chromatography (US Environmental Protection Agency 1997, method 300.1) using a Dionex DX‐120 instrument and a carbonate (2.4 mM)/bicarbonate (3 mM) buffer (pH 10.4) as eluent.

In natural waters TSS was determined by filtering a 200‐mL water sample through a Millipore polyvinylidene fluoride (PVDF) filter 0.45‐µm membrane of known weight, aided by a vacuum pump (GAST; model DDA‐V112‐BN). The material retained, and the filter was dried in a desiccator for 1 wk at 20 °C. When a constant weight was confirmed, the TSS concentration was calculated by the difference in weight. Weight measurements were performed using an analytical scale (Precisa 125 A; Precisa Gravimetrics).

Quantification of Al in TSS was performed by acid digestion of filtered solids and filtered according to USEPA method 3052 (US Environmental Protection Agency 2004) in a mixture of 4.5 mL of HCl 30%, 2 mL of hydrofluoric acid 40%, and 1.5 mL of HNO_3_ 65% (all Merck Suprapur acids). Three replicates of each sample were digested in closed polytetrafluoroethylene vessels assisted by a microwave (model Ethos D; Milestone). Any PVDF filters with no TSS were analyzed using the same protocol with just 200 mL of deionized water passes across the filter to determine background metal levels. Acid‐digested filter samples were left to cool overnight, and the sample volume was adjusted to 1 L in a volumetric flask. The Al concentration was measured in an inductively coupled plasma‐mass spectrometer (ICP‐MS) Perkin Elmer model ELAN 9000.

Dissolved Al concentrations were measured after filtering a 10‐mL water sample through a 0.45‐µm membrane (Millex‐HV, 33 mm, PVDF filters from Merck‐Millipore). Three replicates in conical polypropylene tubes were prepared per sample and acidified with HNO_3_ 65% to a pH of < 2. The samples were stored at 4 °C for less than 1 wk before being measured by ICP‐MS (Perkin Elmer model ELAN 9000) following USEPA method 200.8 (US Environmental Protection Agency 1994a).

Total Al concentrations in samples without suspended solids were measured as acid‐soluble Al (Table 1). For toxicity studies with TSS, only pH 4–extracted and dissolved Al were directly measured, and total Al concentrations were determined by summing the amount of Al in the TSS plus the amount of Al added to the test medium. The Al concentrations contained in natural water TSS were determined by total acid digestion of the TSS in a Milestone Ethos Up microwave oven in closed Teflon vessels. When kaolin was present in the test solutions, total Al was calculated from vendor information summed to the external Al. In controlled experiments, we filtered (using 0.45‐µm PVDF membranes of 47 mm diameter) a known volume of natural water with TSS. Next, the filtered TSS was resuspended in the corresponding volume to achieve the desire concentrations of TSS.

The amount of Al added from the soluble stock solution was added to the TSS Al content to calculate a total Al concentration. When the suspended solids source was kaolin clay, total Al concentration was based on the concentration of soluble Al added and the total Al associated with the kaolin clay content according to the information of the reagent vendor.

In the experiments to determine the pH effect on the extractability of Al from natural waters and in USEPA 84 medium, samples were adjusted to the pH value recommended in the various cited methods or to pH 4 with nitric acid (Merck Suprapur) and agitated at 100 rpm for 1 h in a GFL 1092 incubator at 22 °C. Three 10‐mL samples were extracted and filtered through a 0.45‐µm membrane. Measurement of the final pH of the sample and dissolved Al concentration was performed. Kaolin clay was used as a means of utilizing a separate well‐characterized matrix of highly insoluble Al minerals to simulate artificial TSS.

### Bioavailable Al: pH 4.0

Concentrations of Al in the toxicity test vessels were measured (as well as total and dissolved Al) using a pH 4–extractable determination carried out at time 0 h (before the animals were added to the test solutions) and after 24 h (before the test solutions were renewed). The method consisted of the preparation of a 1 M sodium acetate buffer (2.449 g sodium acetate and 4.7 mL acetic acid, completed to 100 mL with deionized water in a volumetric flask) to achieve a pH of 4 in the buffer. If the prepared buffer was outside a range of 4.0 to 4.2, acetic acid was added in µL aliquots to bring the pH to within the target. Polypropylene cups with 25 mL of the test solution were titrated to pH 4 (pH 4.0–4.2) by addition of 1 M buffer and poured into 50‐mL conical polypropylene tubes. The tubes were capped, agitated by hand, and mixed for 3 h at 22 °C at 100 rpm in an orbital shaker. After incubation, tubes were agitated again by hand, and 10 mL of the supernatant was filtered through a 0.45‐µm PVDF membrane (Millex‐HV; Merck‐Millipore). Samples were acidified to 1% nitric acid (Merck Suprapur) and stored at 4 °C until analysis (by ICP‐MS). Buffer reagents were Merck, proanalysis grade.

### Added Al recovery at pH 4

Recovery of pH 4–extractable Al in natural waters or USEPA 84 medium was measured as described in the section *Bioavailable Al: pH 4.0*. This was performed following the addition of soluble (bioavailable) Al from an acid stock solution to test waters adjusted to pH 6.3 and 8.0. This was done in the absence of buffer (pH 8.0) or the presence of buffer, MES 5 mM (pH 6.3). The pH 4 extractability of Al in the presence of added TSS (collected from the Colorado River) or kaolin clay was studied where several concentrations of added Al were incubated for 3 h at pH 4 with agitation (100 rpm) in a GFL 1092 water bath at 22 °C, followed by 0.45‐µm membrane filtration and measurement of Al.

### Al pH 4 extractability kinetics

To determine the kinetics of extraction of Al over time at pH 4, 1 mg/L of Al (from a stock solution of Al[NO_3_]_3_ × 9H_2_O) was added to USEPA 84 water samples, and the extraction efficiency was followed over time. The effect of incubation pH (6.3 and 8.0) and the presence of TSS and artificial TSS (kaolin clay) was tested in USEPA 84 medium. A volume of 500 mL of the test medium was incubated in a GFL 1092 bath and agitated at 100 rpm and 22 °C. Samples were extracted in triplicate at several time intervals subsequent to spiking the test medium and processed by the pH 4 extraction method.

### Quality assurance

Samples were analyzed for total, pH 4–extracted, and dissolved Al at the Laboratory of Ecotoxicology and Chemistry of Metals at Centro de Investigación Minera y Metalúrgica (CIMM) (Santiago, Chile). For quality assurance and control, one in 10 samples consisting of a standard and a blank sample consisting of deionized water were also measured.

### Al measurement in United States surface waters

An evaluation was made of concentrations of total recoverable Al and TSS for 22 607 sites in natural surface waters across the United States using data from the US Geological Survey's National Water Information System. This was done to establish a relationship between TSS and concentrations of total Al that exceed the USEPA chronic water quality criterion for Al used by most states (87 µg/L). In addition, water samples were collected from 11 rivers and one lake in the United States, several of which are listed as impaired for Al (USEPA, 303d list), and analyzed for total, dissolved, acid‐soluble, and pH 4–extractable Al. Samples were collected in 4‐L bottles filled to zero headspace, placed in a cooler, and shipped to the laboratory for analysis where they were processed immediately and measured within 7 d.

The Al measurements were performed at Oregon State University Aquatic Toxicology Laboratory (Corvallis, OR) using the following methods (Table 1). Total Al: Different methods exist for “total” and “total recoverable,” which entail different methods of acid digestion. For the present evaluation of United States surface waters, “total” Al concentrations, as used in the present study, were determined using unfiltered water samples adjusted to a pH of 1 to 2 with HNO_3_, followed by sample storage for up to 7 d, and then analyzed by ICP‐optical emission spectrometry (OES; US Environmental Protection Agency 1994b). Acid‐soluble Al: An unfiltered sample was adjusted to a pH of 1.65 to 1.85 with 1:1 nitric acid:deionized water, digested for 16 h, filtered through a 0.45‐µm filter (US Environmental Protection Agency 1991), preserved with nitric acid, and analyzed by ICP‐OES. pH 4–extracted Al: Described in the section *Al measurement in United States surface waters*. Dissolved Al: Water samples were filtered through a 0.45‐µm filter, preserved with nitric acid to a pH of 1 to 2, and analyzed by ICP‐OES (US Environmental Protection Agency 1994b).

In addition to the analytical measurements for Al, the surface waters were analyzed for pH (HQ30d pH meter/probe), hardness (standard methods 2340 C; American Public Health Association 2012a), DOC (standard methods 5310B; American Public Health Association 2012b), and TSS (standard methods 2540D; American Public Health Association 2012c). Appropriate quality assurance and quality control samples were utilized (i.e., blanks, duplicate analyses, and standards).

### Statistical analyses

Biological dose responses were observed to correlate with total Al, as discussed by Cardwell et al. (2018) and Gensemer et al. (2018). Dissolved concentrations remained relatively constant in the low µg/L range independent of the increasing test concentrations. Statistical analyses were performed using measured average total Al concentrations.

The effect on reproduction was calculated as the number of neonates produced per adult female (the total number of neonates produced until either the death of the adult or the end of the experiment). No‐observed‐effect concentrations and lowest‐observed‐effect concentrations were calculated using Dunnett's multiple comparison method (EMSL Cincinnati Dunnett Software, Ver 1.5). To estimate concentrations that would cause 10, 20, and 50% reductions in reproduction, expressed as 7‐d EC10, EC20, and EC50, respectively, with corresponding 95% confidence intervals, calculations were performed using a point estimation technique by means of linear interpolation with the USEPA program ICp, Ver 2.0 (Norberg‐King 1993). (Additional details for Table 2 [EC20 values] are available in the Supplemental Data.)

**Table 2:** Toxicity results for *Ceriodaphnia dubia* in natural and artificial waters spiked with 5 concentrations of Al at pH 6.3 and 8

		Studies performed at pH 6.3			Studies performed at pH 8.0		
Media	TSS derived Al (mg/L)^b^	EC50 (mg/L)	EC10 (mg/L)	Survival (%)	EC50 (mg/L)	EC10 (mg/L)	Survival (%)
Simulated Colorado River water, no TSS^c^	0	0.4	0.1	0	1.1	0.5	20
		(0.3–0.4)	(0.0–0.2)	(1.4)	(0.9–1.2)	(0.3–0.6)	(3.3)
Colorado River water, no TSS	0	—	—	—	2.5	0.6	100
					(2.2–2.8)	(0.4–1.0)	(3.4)
Colorado River water with TSS (61 mg/L)	4	—	—	—	2.4	0.9	100
					(2.0–2.8)	(0.1–1.1)	(3.1)
Simulated Colorado River water with TSS (200 mg/L)	22	0.6	0.4	90 (1.4)	—	—	—
		(0.5–0.8)	(0.2–0.4)				
Simulated Colorado River water with kaolin (104 mg/L)	22	1.0	0.5	20 (1.5)	1.4^d^	0.4	80
		(0.9–1.1)	(0.1–0.8)		(1.0–1.8)	(0.1–1.0)	(3.5)

^a^ In the toxicity studies Al was measured as pH 4 extractable Al. The EC50 and EC10 values are calculated for effects on reproduction. Percent survival information is reported for only the highest concentration tested because there was no dose response for survival (in parentheses).

^b^ The TSS‐derived Al concentrations were obtained using the method for total Al.

^c^ Laboratory water was adjusted to match the water quality characteristics of the Colorado River.

^d^ For this test at pH 8, 19.2 mg of kaolin was added containing 4 mg/L mineralized Al. = no test performed; EC10/EC50 = 10% and 50% effect concentrations; TSS = total suspended solids.

## RESULTS

### Effect of TSS on C. dubia survival and reproduction

**Figure 1:**
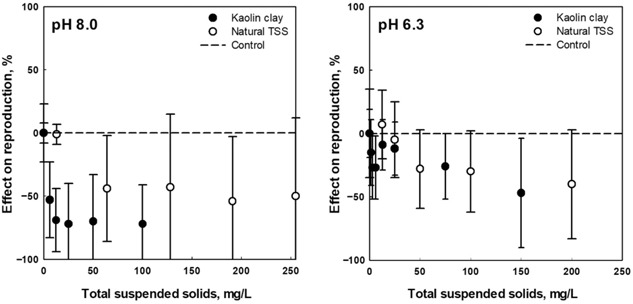
Chronic effects of total suspended solids (TSS) on *Ceriodaphnia dubia* reproduction (day 7) at pH 6.3 and 8.0. Data are presented as a relative decrease in reproduction compared to the controls, where no TSS was present. Natural or artificial TSS (kaolin) were added to the test vessels in increasing concentrations. Closed circles represent the data for kaolin clay (artificial TSS). Open circles represent the relative reproduction obtained with increasing concentration of natural TSS (Colorado River TSS obtained by filtration through 0.45‐μm polyvinylidene fluoride membranes and resuspended in the test media).

The effect of TSS on *C. dubia* survival and reproduction in Colorado River water with increasing concentrations of TSS (2–255 mg/L) was characterized at pH 6.3 and 8.0. The TSS had no effect on the survival of the daphnids. However, 7‐d chronic tests clearly indicated an inhibitory effect of TSS on reproduction, where reproduction was typically reduced by 50% or more with no Al added (Figure 1). The same effects were not seen with filtered water from the same 2 sites (i.e., with TSS removed, reproduction was normal; Supplemental Data, Plot S1). To confirm that the inhibitory effect on reproduction was attributable to the presence of TSS and not to an unknown contaminant, a sample of TSS obtained by filtration from the Tinguiririca River water was resuspended in the same volume of filtered natural water from the Colorado River, where inhibition of reproduction had also been observed (Supplemental Data, Plot S1). Once again, reproduction was reduced because of the presence of the suspended solids, whereas it was not reduced in filtered river water. This effect on reproduction is attributed to an interference in the feeding process of the daphnids and their ability to obtain sufficient nutrition because they are filter‐feeders. This has been widely documented in the literature (Kirk and Gilbert 1990; Kirk 1991; Bilotta and Brazier 2008; Robinson et al. 2010; Arendt et al. 2011; Zhang et al. 2013), where factors like TSS concentration, composition, particle size distribution, and exposure time play a major role in the health of the organisms. Observations of the reproduction performance during the tests suggest that the TSS effect on *C. dubia* may be more important in the first half of the test, with younger, smaller animals.

**Figure 2:**
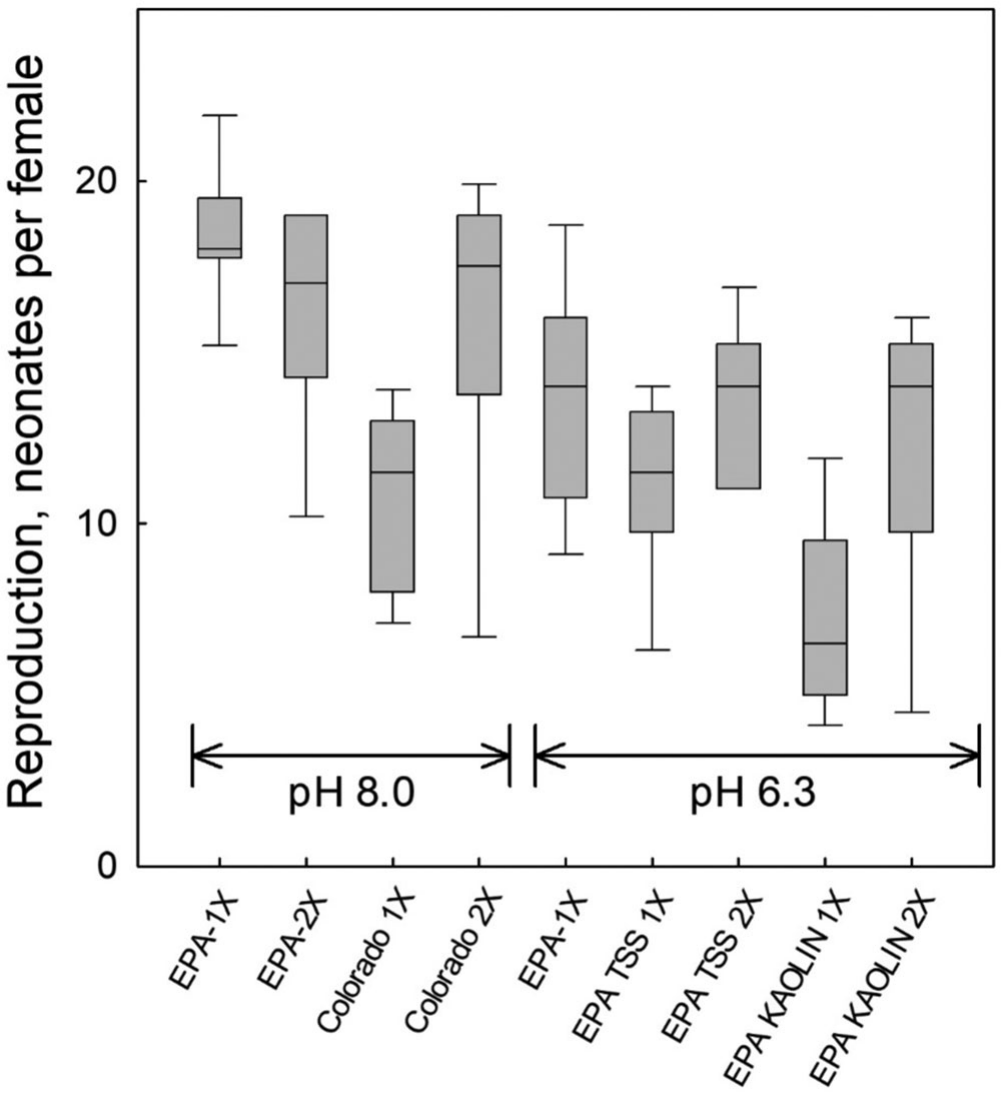
Effect of food concentration on *Ceriodaphnia dubia* reproduction in the presence of natural and artificial total suspended solids (TSS) at pH 8.0 and 6.3. Feeding regimen is shown as “1×” and “2×,” times the normal amount of food. The test media were US Environmental Protection Agency (USEPA) media and Colorado River water. The TSS was added as Colorado River TSS obtained by filtration and resuspended or as kaolin clay. Tests represented as “EPA 1×” contained no TSS and reflect normal test results in the absence of TSS. The TSS in natural Colorado waters (pH 8.0) was 169 mg/L. The TSS in USEPA 1× and 2× (pH 6.3) was 206 mg/L. Kaolin clay in USEPA 1× and 2× (pH 6.3) was 150 mg/L.

Experiments were conducted at pH 6.3 and 8.0 using USEPA medium with kaolin added and Colorado River water with TSS with food additions at normal levels (1×) and twice the normal amount (2×). The results indicated that daphnid reproduction was reduced in the presence of TSS, with the number of neonates per female ranging from 6 to 11 (mean = 9.3) in tests with TSS and 1× normal food (Figure 2). Reproduction in tests with 2× the amount of food showed an improvement, and reproduction ranged from 14 to 18 (mean = 15.3) neonates per female. Reproduction in test media with no TSS and a normal amount of food ranged from 14 to 18 and averaged 16 neonates per female. A reproduction level of 15 neonates per female is frequently used as a criterion for acceptability of the test when used for regulatory purposes.

### Toxicity of Al in waters naturally high in TSS

**Figure 3:**
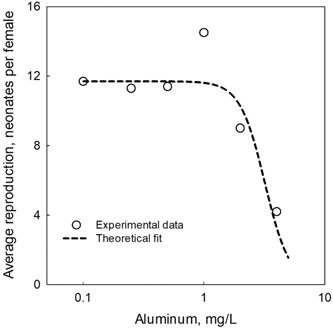
*Ceriodaphnia dubia* reproduction dose–response effect of added bioavailable Al in natural waters containing 61 mg/L of total suspended solids (TSS; Al content in the TSS, 3.8 mg/L). Reproduction of *C. dubia* is plotted as a function of the increase in bioavailable Al (added to the media) on a log scale. Dashed line represents the curve fit of the log‐logistic dose–response equation to the experimental data points.

Colorado River water (pH 8.0), with elevated TSS (61 mg/L and total Al 3.8 mg/L), was spiked with increasing concentrations of soluble/bioavailable Al, in the form of Al(NO_3_)_3_ × 9H_2_O. Effects on survival and reproduction were measured and indicate that the added Al significantly reduced reproduction, demonstrating that metal toxicity could be observed in the presence of TSS. An EC50 of 3.2 mg bioavailable Al/L was calculated based on reproduction (Figure 3).

### Ability of acid extraction to differentiate bioavailable Al in natural waters from mineralized particulate forms of Al

**Figure 4:**
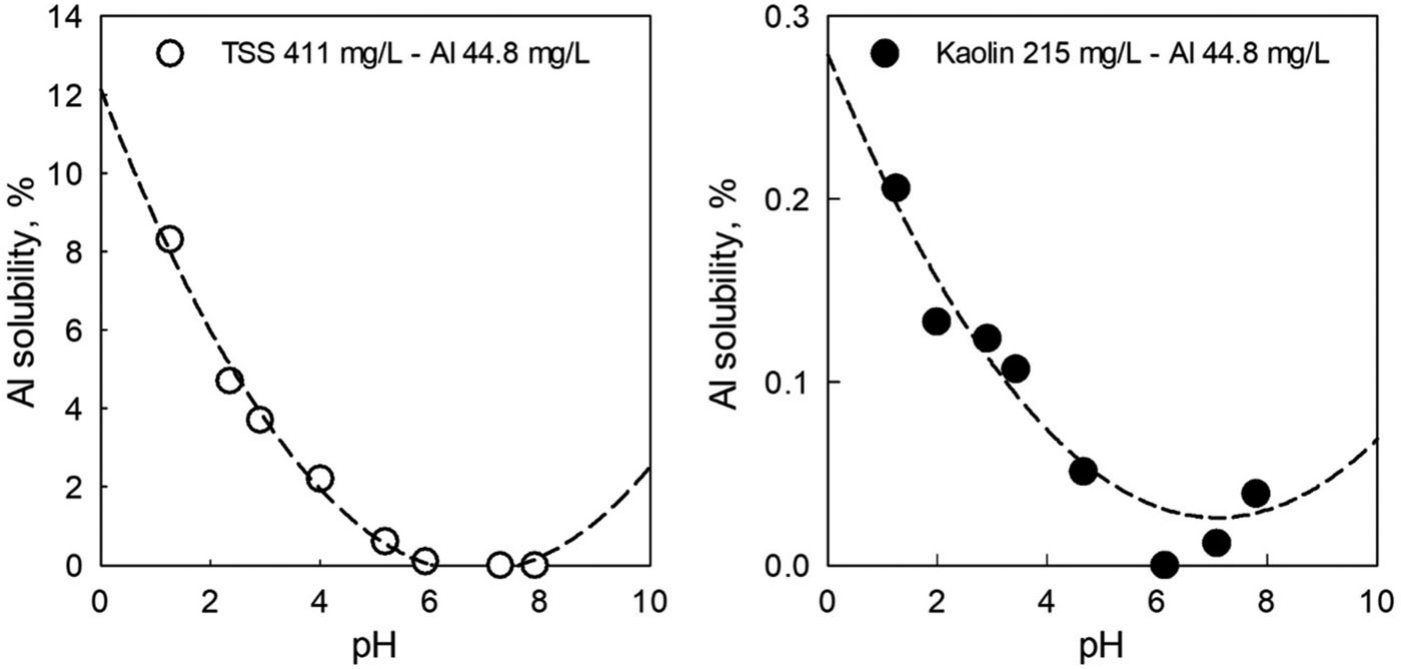
Aluminum dissolution from waters with a high content of natural and artificial total suspended solids (TSS). Natural Colorado River waters with 411 mg/L TSS (total Al content 44.8 mg/L) and US Environmental Protection Agency 84 media with 215 mg/L kaolin clay (artificial TSS) and the same total Al concentration were incubated for 1 h at different pH values, filtered, and analyzed for Al. Dissolved Al was expressed as a percentage of the total Al present in the test. The pH values were obtained by adjusting the water samples with HNO_3_.

Tests were performed to evaluate the importance of the strength of the acid digestion on recovery of Al and to distinguish between bioavailable Al and mineralized forms of the Al metal present in test waters with suspended solids. An Al acid extraction test was performed in water from the Colorado River (natural TSS) and in USEPA 84 medium in the presence of artificial TSS (kaolin clay). The water samples had equal amounts of total Al (44.8 mg/L). The amount of Al dissolution from the TSS varied inversely with increasing pH for both water and TSS types. The resulting concentrations of total Al in solution ranged from 0.04 to 3.71 mg/L, which was equivalent to <0.05 to >8% of the total Al present in the TSS (Figure 4). Note, none of the extractions in this experiment recovered 44.8 mg/L because the extraction conditions were not as rigorous as described in the USEPA method for total recoverable Al.

Extraction of total Al from the 2 types of TSS resulted in an order of magnitude more Al released from the natural TSS than from artificial TSS (kaolin clay; Figure 4). This reflects the strong binding sites associated with kaolinite attributable to the crystalline arrangement of the atoms within the mineral (Robinson et al. 2010). In contrast, natural TSS may contain multiple mineral phases, including clay minerals such as illite, smectite, and chlorite, which are more easily digested by acid (Manassero et al. 2008; Abdullayev and Leroy 2016). Nevertheless, the data show that the amount of Al extracted decreases at higher pH values, with a majority of the Al contained in the nonsoluble phases within the TSS particles. These data provided the basis for evaluating an extraction pH that would recover Al that is dissolved or loosely associated with particles.

To obtain an appropriate discrimination between bioavailable and mineralized forms of Al, an extraction pH that minimizes the solubilization of mineralized forms and maximizes the extraction of bioavailable forms of Al was desired. Selection of a pH value of 4 for the extraction is a compromise in acid strength selection designed to avoid extracting most of the Al in the mineral phases but strong enough to not lose the bioavailable Al. The main difference between the current proposed pH 4 Al extraction method and the USEPA acid‐soluble method (or total recoverable Al) is the pH value chosen for the metal extraction. The USEPA acid‐extractable protocol adjusts the sample to pH 1.5 to 2.0 with nitric acid. On the other hand, the present protocol used pH 4 with a 1 M sodium acetate buffer and a 3‐h extraction period. Extraction at pH 4 was selected based on preliminary experiments where pH was varied.

### Aluminum pH 4 extraction kinetics

**Figure 5:**
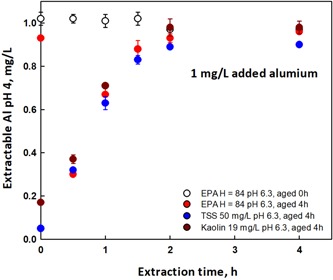
Dissolution kinetics of Al extracted at pH 4 from samples of US Environmental Protection Agency 84 media at pH 6.3 and aged 0 and 4 h in the presence or absence of natural and artificial total suspended solids (TSS). The media had 1 mg/L Al added. Both natural and artificial TSS have 5 mg/L total Al content and with the addition of 1 mg/L Al from a nitrate salt reached a total Al content of 6 mg/L. EPA = US Environmental Protection Agency.

To evaluate the length of time needed for the extraction period, the dissolution kinetics of Al extracted at pH 4 from samples of USEPA 84 medium at pH 6.2, in the presence and absence of natural and artificial TSS, were measured at several time intervals. The data show that complete recovery of the Al added could be achieved in 3 h (Figure 5). It can also be seen that at time zero the total and pH 4 methods both recovered similar amounts ( >90%) of added Al (1 mg/L) in the absence of TSS. In the presence of TSS, the pH 4 extraction recovered 90% or more after 3 h, with no additional recovery at 4 h.

### Dissolution of Al: pH 4 extraction versus EPA pH 1.5 procedure

**Figure 6:**
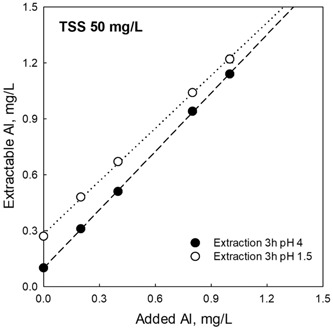
Comparison of 2 extraction procedures (pH 1.5 and pH 4) using US Environmental Protection Agency media and an extraction period of 3 h. The test media used for the extraction contained 50 mg/L of natural total suspended solids containing 5 mg/L total Al. Five concentrations of Al were evaluated, ranging between 0 and 1 mg/L. The amount of Al extracted at various time periods is presented; filled circles represent the pH 4 procedure, and empty circles represent the pH 1.5 procedure. TSS = total suspended solids.

To assess the dissolution of Al in USEPA medium at 3 h with and without TSS (50 mg/L), extractions were performed over time to compare the results of the pH 4 method with the USEPA pH 1.5 extraction procedure. The results indicate that both protocols attained good recoveries of added Al, with > 90% of the added Al recovered (Figure 6). However, extraction at pH 1.5 produced more metal than was added, indicating that Al was released from the mineral phases. The data demonstrate that strong acid extraction leads to an overestimation of the amount of Al that was added. The inference here is that a procedure is needed that is less rigorous to measure only the Al that is bioavailable.

### Dose–response relationship between toxicity and pH 4–extractable Al

Several chronic bioassays performed with *C. dubia* showed a dose–response relationship between pH 4–extracted Al concentrations and inhibition of reproduction (see *Methods* section for the design). To obtain TSS for testing, natural water samples containing TSS were filtered to obtain the solid fraction, and then the filtered material was resuspended in the appropriate volume of test water to reach 200 mg/L containing 22 mg/L of mineralized Al. Effect concentration values are reported with 95% confidence intervals (Table 2). Test results for these experiments show that daphnids exposed to soluble Al in the presence of elevated levels of mineralized Al (4–22 mg/L) resulted in EC50 and EC10 values similar to the tests where there was no mineralized Al (tests 1 and 2, Table 2). Hence, the mineralized Al appears to contribute little to the toxicity. Expressing the toxicity values on a total Al basis would be erroneous because of the presence of TSS in the studies.

To further illustrate the difference in reporting toxicity results based on total Al versus pH 4–extractable Al, 7‐d chronic EC50 values are shown in Table 3 using measured pH 4 and total Al concentrations (total Al concentrations reflect the amount of Al in the TSS plus the Al added to the test solution). The information presented shows that EC50 values measured as pH 4 Al are reasonably consistent (pH 6.3, 0.4–1.0 mg/L; pH 8.0, 1.1–2.5 mg/L), whereas the EC50 values reported as total Al are considerably higher, variable, and not related to the amount of Al added to the test solution. The key point here is that the EC50 values measured in the presence of TSS closely resemble those measured with no TSS, indicating that the pH 4 method measures predominantly bioavailable Al and ignores the particulate Al.

**Table 3:** Toxicity results for *Ceriodaphnia dubia* reproduction in natural and artificial waters spiked with 5 concentrations of Al at pH 6.3 and 8^a^

Test no.	TSS (mg/L)	Total Al in TSS (mg/L)	EC50 at pH 6.3 measured using a pH 4 extraction (mg/L)	EC50 at pH 6.3 reported as total Al (mg/L)	EC50 at pH 8.0 measured using a pH 4 extraction (mg/L)	EC50 at pH 8.0 reported as total Al (mg/L)
1	0	0	0.4	0.4	1.1	1.1
2	0	0	—	—	2.5	2.5
3	61	4	—	—	2.4	6.4
4	200	22	0.6	22.6	—	—
5	104	22	1.0	23.0	1.4	23.4

^a^ EC50 values are reported as pH 4 extractable Al values and based on total in the test media.

EC50 = 50% effect concentration; TSS = total suspended solids.

**Figure 7:**
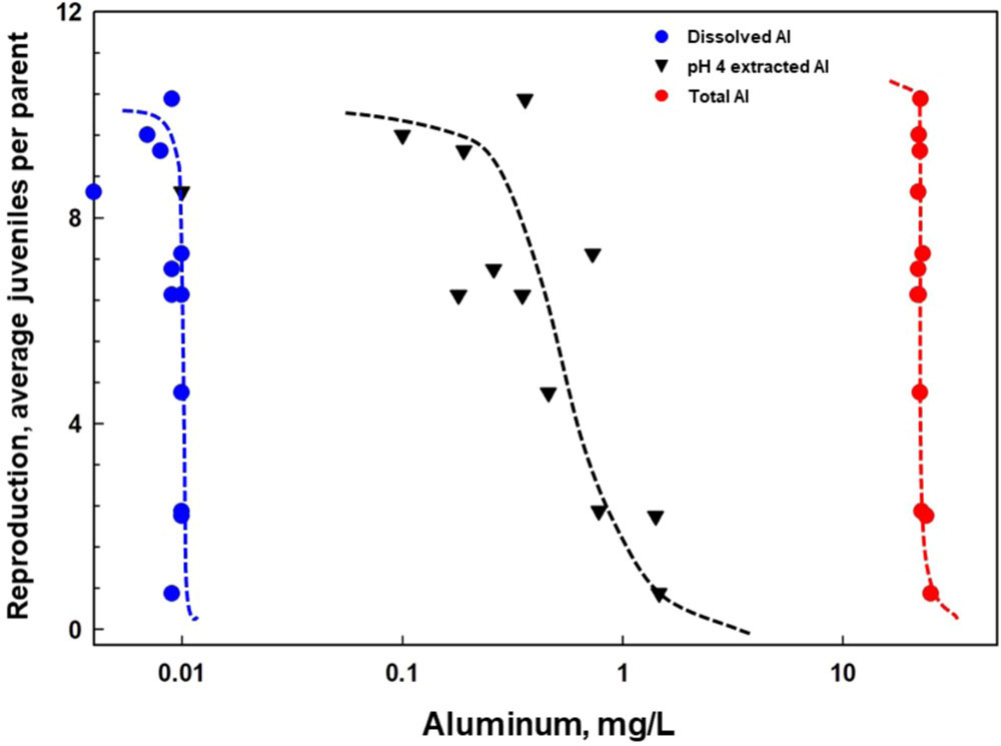
Comparison of *Ceriodaphnia dubia* reproductive effects as a function of Al concentration measured as dissolved, pH 4, and total Al. Two toxicity tests are combined in the figure; both were performed at a pH of 6.3 in the presence of natural total suspended solids (200 mg/L) containing 21.8 mg total Al/L and kaolin (104 mg/L) containing 22 mg total Al/L.

To demonstrate the inability of either dissolved or total Al to accurately measure reproductive effects of Al on *C. dubia*, the results of 2 toxicity tests in the presence of TSS are jointly presented in one figure (Figure 7), where the Al concentrations were measured as dissolved, pH 4–extracted, and total Al. The figure clearly shows that only the pH 4 measurements accurately measured the toxicity. The data for Figure 7 are contained in Supplemental Data, Tables S8 through S11. A comparison of the nominal concentrations in these 2 tests with the pH 4 measurements indicates that the average percent of nominal is 80% (Supplemental Data, Table S12), further supporting the ability of this analytical method to recover the Al that was added in the presence of significant levels of TSS.

### Toxicity assessment using the pH 4 acid extraction method with several Al compounds

To assess the relationship between toxicity and extracted Al using the pH 4 method, several Al compounds with a range of solubilities were independently assayed using C. dubia in Colorado River water with 30 mg/L TSS that contributed 2 mg/L mineralized Al when measured as total Al. The added Al concentrations of 2.7 to 4.0 mg/L were selected to enable observation of a reduction in reproduction of approximately 50% (EC50; Table 4). The toxicity data indicate that only Al compounds with a high degree of solubility (i.e., Al nitrate and sodium aluminate, which are easily extracted at pH 4) have an inhibitory effect on C. dubia reproduction. The pH 4 method extracted very little of the Al from insoluble forms of Al (Al oxide and atomized Al), and these forms were nontoxic, thus confirming that the extracted Al fraction is responsible for the observed toxicity and supporting the view that pH 4 extraction is a better approach to assessing bioavailable Al than total Al.

**Table 4:** Chronic toxicity of several forms of Al to *Ceriodaphnia dubia* in Colorado River water containing 2 mg/L mineralized Al derived from total suspended solids (TSS)^a^

Compound^b^	Added Al (mg/L)	Al pH 4 extracted (mg/L)	Live adults	Offspring per adult	Standard deviation	pH
Colorado River water	0.0	0.02	10	12.8	4.1	8.25
Al(NO_3_)_3_ × 9H_2_O	4.3	3.9	10	4.8	1.5	8.26
Sodium aluminate	3.5	3.5	10	3.6	1.2	8.27
Aluminum oxide (Al_2_O_3_)	2.7	0.03	10	12.7	4.3	8.27
Atomized aluminum	4.0	0.25	10	13.7	3.1	8.27

^a^ The river water was supplemented with different Al compounds at one concentration close to previously determined Al EC50 values at pH 8.0 (i.e., ~3 mg/L). Tests were performed with the standard supply of food. The Al in the test vessels was measured as pH 4 extracted Al after being in the test chamber for 24 h. The Al associated with the TSS was measured as total Al.

^b^ Aluminum nitrate nonahydrate (Merck, Chemical Abstracts Service no. 7784‐27‐2): Al(NO_3_)_3_. Nine H_2_O; sodium aluminate (Riedel‐de Haën, European Inventory of Existing Commercial Chemical Substances no. 235‐487‐0); aluminum oxide, SO 143 (aluminum oxide stade) and atomized aluminum granules ECKA (Al 99.5% AS < 10 µm).

EC50 = 50% effect concentration; AOS = aluminium oxid stade.

### Al concentrations in United States surface waters

**Figure 8:**
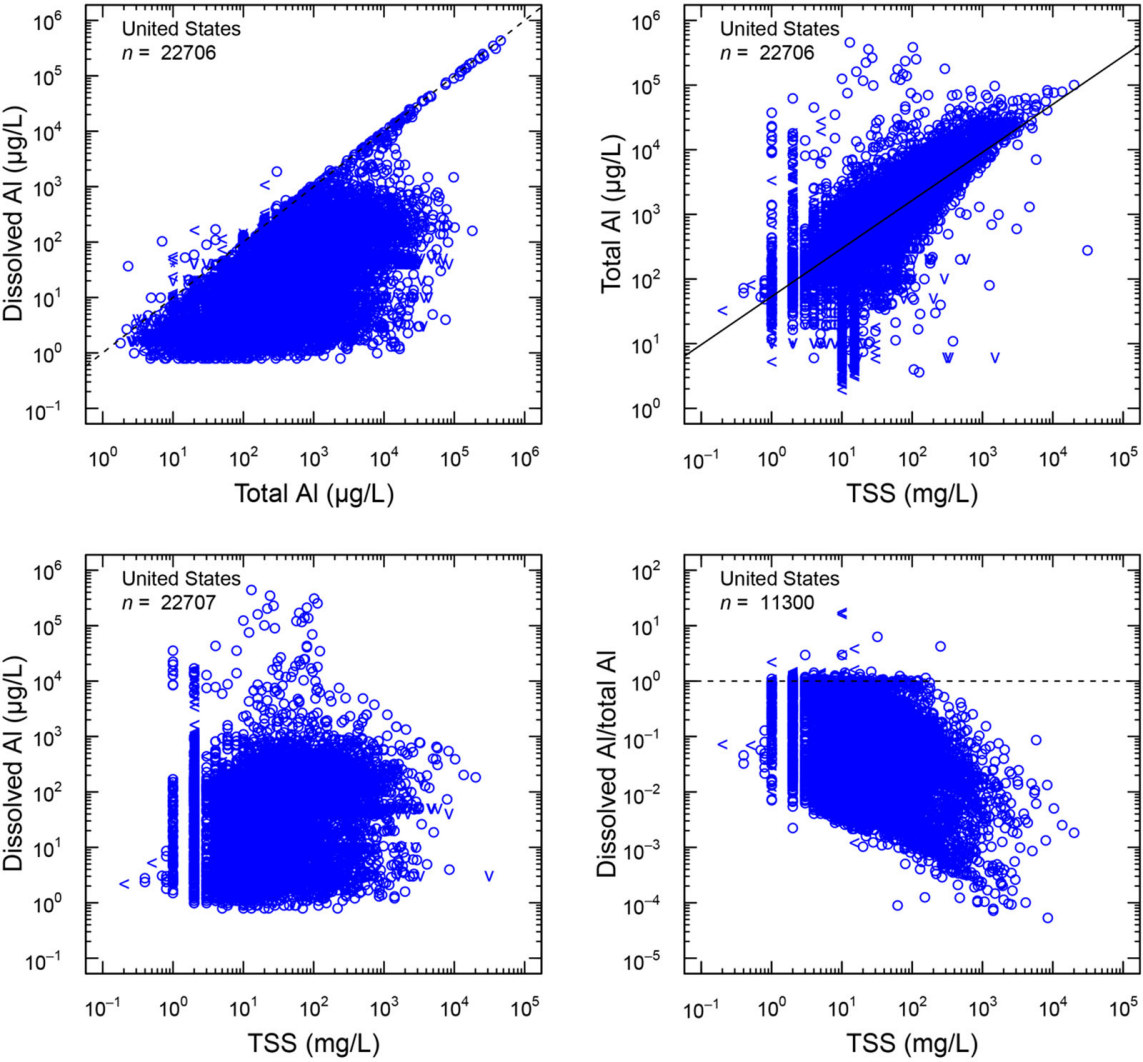
Comparisons of measured Al with total suspended solids in United States surface waters (graphic courtesy of Adam Ryan, Windward Environmental). TSS = total suspended solids.

An evaluation was made of surface water concentrations of total (recoverable) Al and TSS in natural waters across the United States using data from the US Geological Survey's National Water Information System. The relationship between total Al and TSS based on 22 607 samples is easily observed (Figure 8). However, there was little or no relationship with dissolved Al. The data in Figure 8 (top right panel) show that a large percentage of the national surface waters do not meet the chronic water quality standard (87 µg/L). It is clear from this analysis that the Al being measured as total Al is, for the most part, being derived from the TSS. It is not likely that this Al would be bioavailable because strong acid digestion was required to release the Al. The present study demonstrates that Al in TSS is not bioavailable.

To further emphasize the importance of the issue, data are presented on 11 rivers and one lake in the United States (Table 5). The data show that the total Al concentrations in these rivers are quite high, ranging from 5 to 3740 µg/L, and the suspended solids ranged from < 1 to 624 mg/L. These rivers were specifically selected to cover a wide range of TSS, DOC, hardness, and pH that might exist across the United States and Europe. The inference here is that the majority of the Al is from the suspended solids and that the Al concentration when measured as total Al frequently exceeds the current USEPA National Ambient Chronic Water Quality Criterion for Al (87 µg/L). Eight of the 12 natural waters evaluated do not meet the USEPA water quality criteria. Dissolved Al measurements were quite low, often <20 µg/L. The scientific literature shows that dissolved Al does not correlate with Al toxicity to aquatic organisms (Cardwell et al. 2018; Gensemer et al. 2018). Concentrations of Al determined using the pH 4 method were consistently less than total or acid‐soluble Al and were greater than dissolved measurements. The highest to lowest order of Al concentrations was total > acid‐soluble > pH 4 > dissolved (Table 5).

**Table 5:** Concentrations of total, acid‐soluble, pH 4 extractable, pH 2, and dissolved Al in US surface waters for the purpose of comparing the pH 4 method to other analytical measures of Al

		Surface water locations in the United States											
	Parameter	Lookout Creek, OR	Mack Creek, OR	Tahquamenon River, MI	Pine River, MI	Carp River, MI	Mississippi River, MO	Bear River, UT	N. Santiam River, OR	Zollner Creek, OR	Luckiamute River, OR	Coffenbury Lake, OR	Ohio River, OH
	TSS	0.07	0.18	2.1	2.2	2.3	13.4	46	142	249	177	219	624
	pH	7.1	7.1	7.1	7.8	7.8	8.1	8.2	7.	7.1	6.6	6.0	7.8
	Hardness (mg/L)	12	12	56	140	140	236	236	16	124	20	30	148
	DOC (mg/L)	0.63	0.65	17.5	10.4	9.86	5.61	2.60	2.56	7.98	3.54	19.4	3.73
Al concentrations (μg/L)													
	Total Al (μg/L)	17	3.8	179	506	40.5	245	323	2772	3197	2393	2315	3740
	Acid‐soluble Al (μg/L) (pH 2)	5.2	7.5	120	130	30	46	90	548	562	686	1761	991
	pH 4 extracted Al (μg/L)	<0.2	1.4	96	45	14	<0.2	10	36	102	50	496	247
	Dissolved Al (μg/L)	<0.2	1.0	90	31	15	<0.2	< 3.0	19	4	28	439	5.3

TSS = total suspended solids; DOC = dissolved organic carbon.

Recently, the USEPA has updated it water quality criteria for Al using a multiple linear model approach based on the work of DeForest et al. (2018) to calculate the criterion for a given site‐specific water chemistry (US Environmental Protection Agency 2018). This will allow for the criterion to increase for waters with elevated DOC, hardness, and pH in the 7 to 9 range. However, water samples high in TSS will still fail to meet the criterion because of the use of total Al as the analytical method.

## DISCUSSION

The relationship between Al and toxicity presents a dilemma in terms of how to most appropriately measure bioavailable Al in natural surface waters to assess potential for toxicity. Surface waters typically contain suspended solids that naturally contain Al oxides and/or silicates. Analytical determinations using strong acid digestions result in most or all of the aluminum present in inert sediment particles being dissolved and the metal reported as “total” or “total recoverable.” This is in contrast to water samples analyzed in laboratory toxicity tests, which are devoid of suspended minerals. In waters with elevated suspended solids the Al contributed from the solids can result in Al concentrations that significantly exceed the USEPA chronic water quality criterion used by most states (87 µg/L) and will likely exceed the revised Al water quality criteria based on an approach using multiple linear regression (DeForest et al. 2018) for surface waters low in pH, DOC, and hardness and elevated TSS. As a consequence, many United States waters are listed as “impaired for Al” because of the use of the total recoverable or acid‐soluble analytical method. This designation implies that these waters may result in deleterious effects on aquatic life when in fact the Al concentrations are a product of aggressive digestion procedures that overestimate the bioavailability of the metal. Therefore, designating such waters as impaired based on total Al measures may be misleading.

The present study provides evidence that natural waters often contain elevated levels of Al when the samples are unfiltered and measured as total Al. The present laboratory toxicity studies with C. dubia show that the waters high in total Al may not be toxic because much of the Al is not bioavailable. We have demonstrated that C. dubia can survive and reproduce in the presence of elevated levels of TSS and, hence, was useful for assessing the bioavailability of Al in the presence of solids. The TSS does interfere with the ability of the test species to obtain sufficient nutrition for reproduction, but this can be overcome by increasing the food content during the toxicity test. Further work is now in progress with natural waters and fish to demonstrate the usefulness of the pH 4 extraction procedure. A key question that needed to be answered was whether the toxicity of Al can be measured in the presence of high TSS and correlated with the analytical method used to measure the Al concentration. The present study shows that when Al is added to natural waters high in TSS, toxicity can be measured and explained using an extraction of the Al at a pH of 4. In the same studies no relationship was observed with either total or dissolved Al.

## CONCLUSION

In summary, an acid extraction analytical method at pH 4 was developed and tested. The proposed method is better able to discriminate between the chronic effects of bioavailable/toxic and mineralized nontoxic forms of Al to C. dubia compared with existing methods using total or acid‐soluble Al (i.e., extraction at pH < 2). Additional work is in progress to evaluate the method with a broader range of species in natural waters using pH 4 extraction to compare bioavailable Al with dissolved and total Al. It is proposed that this method be used in evaluating compliance with water quality criteria or environmental quality standards.

## Supplemental Data

The Supplemental Data are available on the Wiley Online Library at DOI: http://10.1002/etc.4448.

## Supporting information

This article includes online‐only Supplemental Data.

Supporting information

## Data Availability

Please contact the corresponding author, William Adams, or Patricio H. Rodriguez for any meta‐data or calculations not already provided as Supplemental Data.
