# Supplementary material for: Determination of Bioavailable Aluminum in Natural Waters in the Presence of Suspended Solids
Source: Environ Toxicol Chem. 2019 Jul 25;38(8):1668–81. doi: 10.1002/etc.4448 (PMC6852577; doi:10.1002/etc.4448)
Supplement: Supplementary file 1 — Supporting information [file ETC-38-1668-s001.docx]

**Supplementary Material**

**Chemical characterization of Colorado and Tinguiririca rivers waters**

Two rivers carrying a high content of suspended material, were. The first river “Colorado” is located in the Metropolitan region close to the Chilean capital, Santiago. To avoid any anthropogenic contribution in the water shed, the river was sampled high in the mountains, in the coordinates 33°30’36.6’’ S – 70°12’17.7’’ W. The second river “Tinguiririca” is located in Chilean VI region, more than a 100 km South of Santiago. This river also was sampled high in the mountains to avoid anthropogenic contributions; in coordinates 34°44’23.2’’ S – 70°45’10.7’’ W. A complete characterization of both river waters was performed. Measured chemical parameters are presented in table S1 and S2.

**Table S1-A.** Characterization of water chemical parameters for Colorado river sampling campaigns.

| **Parameter** | **Colorado**  **2-4-2016** | **Colorado**  **4-14-2016** | **Colorado**  **6-16-2016** | **Colorado**  **7-7-2016** | **Colorado**  **12-21-2016** |
| --- | --- | --- | --- | --- | --- |
| pH | 7.90 | 7.87 | 7.63 | 8.19 | 8.04 |
| Conductivity, µS | 878 | 1187 | 1081 | 1189 | 616 |
| TDS, mg/L | 440 | 594 | 540 | 594 | 308 |
| TSS, mg/L | 255 | 169 | 61 | 30 | 411 |
| Alkalinity as mg/L CaCO_3_ | 74.4 | 90.0 | 89.7 | 99.0 | 66.6 |
| Hardness as mg/L CaCO_3_ | 370.3 | 425.0 | 364.5 | 407.9 | 299.8 |
| DOC, as mg/L C | 0.3 | 0.3 | 0.3 | 0.2 | - |
| Phosphorus, µM | < 0.5 | < 0.2 | < 0.5 | < 0.3 | < 0.2 |
| Chloride, mg/L | 52.0 | 134.8 | 99 | 111.3 | 35.8 |
| Sulfate, mg/L | 273.9 | 301.8 | 212 | 248.3 | 249.9 |
| Nitrate, mg/L | < 3 | < 3 | < 3 | < 3 | < 3 |
| Sodium, mg/L | 38.0 | 91.1 | 85.8 | 89.0 | 21.8 |
| Potassium, mg/L | 2.3 | 3.2 | 2.9 | 3.4 | 2.3 |
| Calcium, mg/L | 112.5 | 114.3 | 94.4 | 114.3 | 85.8 |
| Magnesium | 14.6 | 14.4 | 17.6 | 15.5 | 10.5 |

**Table S1-B.** Metals characterization of water from Colorado river sampling campaigns.

| **Dissolved Metal, µg/L** | **Colorado**  **2-4-2016** | **Colorado**  **4-14-2016** | **Colorado**  **6-16-2016** | **Colorado**  **7-7-2016** | **Colorado**  **12-21-2016** |
| --- | --- | --- | --- | --- | --- |
| Fe | < 50 | < 50 | < 50 | < 50 | 312 |
| Co | 0.21 | 0.24 | 0.22 | 0.40 | 0.18 |
| Mn | 22.9 | - | 10.4 | 9.4 | 20.0 |
| Zn | < 20 | < 20 | < 20 | < 20 | < 10 |
| Mo | 4 | - | 5 | 4 | 4 |
| Al | 28 | 15 | 15 | 19 | 19 |
| Cu | < 1 | 1.2 | 1.3 | 1.1 | < 1 |
| Cd | < 0.05 | < 0.05 | < 0.05 | < 0.05 | < 0.01 |
| Ni | 3 | 4 | 4 | 4 | 3 |
| As | 7.7 | 8.9 | 9.2 | 9.8 | 5.2 |
| Pb | < 0.5 | 0.9 | < 0.1 | 0.1 | < 0.5 |
| **Particulated Metal, µg/L** | **Colorado**  **2-4-2016** | **Colorado**  **4-14-2016** | **Colorado**  **6-16-2016** | **Colorado**  **7-7-2016** | **Colorado**  **12-21-2016** |
| Fe | 4800 | 4600 | 2600 | 900 | 11590 |
| Co | 2.64 | 1.69 | 1.00 | 0.62 | 1.4 |
| Mn | 168 | - | 55.6 | 32.4 | - |
| Zn | 23 | < 20 | < 20 | < 20 | < 100 |
| Mo | < 1 | - | 1 | < 1 | < 20 |
| Al | 4800 | 6900 | 3800 | 2000 | 44800 |
| Cu | 13 | 4.5 | 5.3 | 3.2 | 29.3 |
| Cd | < 0.1 | < 0.1 | < 0.1 | < 0.1 | < 1 |
| Ni | 3 | 2 | 1 | 1 | < 10 |
| As | 9.8 | 5.6 | 3.5 | 23.9 | 3.8 |
| Pb | < 1 | 1.8 | 1.0 | 0.7 | 33 |

**Table S2-A.** Characterization of water chemical parameters for Tinguiririca river.

| **Parameter** | **Tinguiririca**  **2-4-2016** |
| --- | --- |
| pH | 8.09 |
| Conductivity, µS | 211 |
| TDS, mg/L | 106 |
| TSS, mg/L | 237 |
| Alkalinity as mg/L CaCO_3_ | 16.7 |
| Hardness as mg/L CaCO_3_ | 75.6 |
| DOC, as mg/L C | 0.1 |
| Phosphorus, µM | < 0.5 |
| Chloride, mg/L | 5.9 |
| Sulfate, mg/L | 57.3 |
| Nitrate, mg/L | 0.5 |
| Sodium, mg/L | 7.2 |
| Potassium, mg/L | 1.3 |
| Calcium, mg/L | 22.3 |
| Magnesium | 2.9 |

**Table S2-B.** Metals characterization of water from Colorado river.

| **Dissolved Metal, µg/L** | **Tinguiririca**  **2-4-2016** |
| --- | --- |
| Fe | < 50 |
| Co | 0.12 |
| Mn | 72.4 |
| Zn | < 20 |
| Mo | 1 |
| Al | 27 |
| Cu | < 1 |
| Cd | < 0.05 |
| Ni | 1 |
| As | 1.3 |
| Pb | < 0.5 |
| **Particulate Metal, µg/L** | **Tinguiririca**  **2-4-2016** |
| Fe | 9800 |
| Co | 4.09 |
| Mn | 176 |
| Zn | 29 |
| Mo | 2 |
| Al | 8800 |
| Cu | 25 |
| Cd | 0.21 |
| Ni | 4 |
| As | 10.8 |
| Pb | 7 |

**Plot S1.** Effect of the presence of TSS in the reproduction of *C. dubia*. Box plot shows the range of 25 – 75th percentile of the data (10 replicas) and the median reproduction in each condition. Lines associated with each box correspond to the range of 5 – 95th percentile.


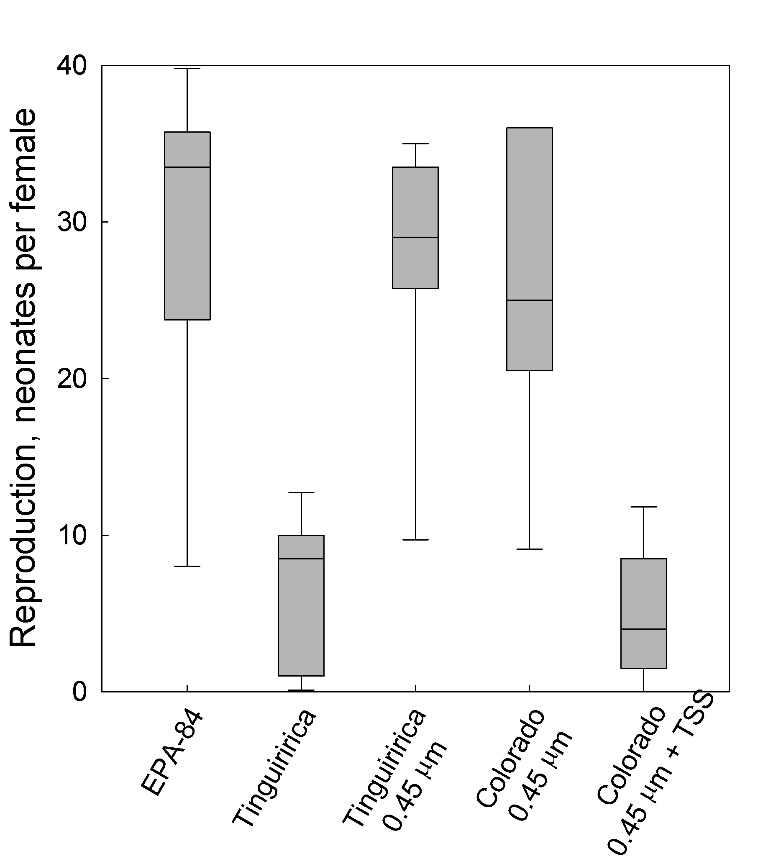


**Plot S2.** Dissolution kinetics of Al extracted at pH 4 from samples of EPA 84 media at pH 6.2 and aged 0 and 4 hours in the presence and absence of natural and artificial TSS. The media had 1 mg/L Al added. Both, natural and artificial TSS have 4-5 mg/L total aluminum content, and with the addition of 1 mg/L Al from a nitrate salt reach a total content of 5-6 mg/L.


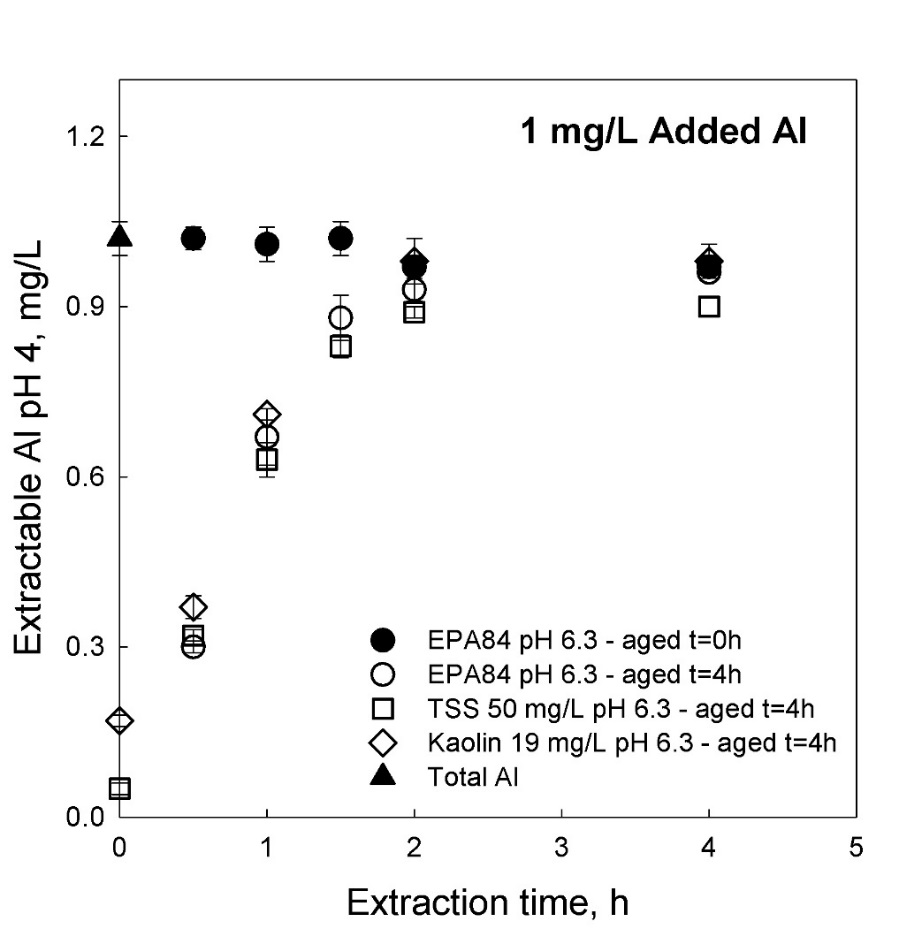


**Table S3 –**Summary of the *Ceriodaphnia dubia* 7 days survival and reproduction Al toxicity test results with simulated Colorado river water (no TSS, hardness 341 mg/L as CaCO_3_, alkalinity 149 mg/L as CaCO_3_, pH 8) and normal feeding. Aluminum was measured as pH 4 extracted Al on 24h old solutions after media renewal, was reported. Toxicity endpoints EC_50_ = 1.1 (0.9 – 1.2) mg/L and EC10 = 0.5 (0.3 – 0.6) mg/L.

| pH 4 - Al  (mg/L) | Number of juveniles per adult replicate | | | | | | | | | | Average  Juveniles | Live  Adults | pH |
| --- | --- | --- | --- | --- | --- | --- | --- | --- | --- | --- | --- | --- | --- |
|  | 1 | 2 | 3 | 4 | 5 | 6 | 7 | 8 | 9 | 10 |  |  |  |
| 0.000 | 16 | 15 | 18 | 16 | 16 | 18 | 18 | 16 | 13 | 19 | 16.5 | 10 | 8.36 |
| 0.173 | 18 | 15 | 21 | 18 | 19 | 12 | 14 | 16 | 15 | 16 | 16.4 | 10 | 8.34 |
| 0.416 | 14 | 8 | 18 | 28 | 19 | 17 | 18 | 17 | 13 | 20 | 17.2 | 10 | 8.34 |
| 0.908 | 13 | 8 | 7 | 12 | 14 | 10 | 8 | 14 | 7 | 6 | 9.9 | 10 | 8.33 |
| 1.633 | 1 | 2 | 4 | 0 | 4 | 3 | 2 | 0 | 0 | 1 | 1.7 | 8 | 8.32 |
| 3.284 | 0 | 0 | 0 | 0 | 0 | 0 | 0 | 0 | 0 | 0 | 0.0 | 2 | 8.32 |

**Table S4 –**Summary of the *Ceriodaphnia dubia* 7 days survival and reproduction of the aluminum toxicity test results with simulated Colorado river water at pH 8 plus kaolin clay (19.2 mg/L) with normal feeding. Aluminum was measured as pH 4 extracted Al on freshly prepared test solutions (time = 0 h) and 24h old solutions after media renew, was reported. Toxicity endpoints EC_50_ = 1.4 (1.0 -1.8) mg/L and EC10 = 0.4 (0.1 – 1.0) mg/L.

| pH 4 - Al  (mg/L) | Number of juveniles per adult replicate | | | | | | | | | | Average  Juveniles | Live  Adults | pH |
| --- | --- | --- | --- | --- | --- | --- | --- | --- | --- | --- | --- | --- | --- |
|  | 1 | 2 | 3 | 4 | 5 | 6 | 7 | 8 | 9 | 10 |  |  |  |
| 0.01 | 8 | 12 | 5 | 11 | 8 | 8 | 3 | 4 | 10 | 7 | 7.6 | 10 | 8.10 |
| 0.22 | 7 | 5 | 10 | 4 | 7 | 2 | 7 | 10 | 10 | 8 | 7.0 | 10 | 8.08 |
| 0.41 | 7 | 6 | 2 | 3 | 9 | 6 | 8 | 12 | 9 | 7 | 6.9 | 10 | 8.07 |
| 0.89 | 4 | 9 | 7 | 3 | 5 | 4 | 6 | 9 | 10 | 2 | 5.9 | 10 | 8.08 |
| 1.71 | 2 | 0 | 3 | 0 | 4 | 6 | 3 | 2 | 4 | 2 | 2.6 | 8 | 8.07 |
| 3.53 | 2 | 0 | 0 | 0 | 1 | 0 | 1 | 1 | 0 | 0 | 0.5 | 8 | 8.06 |

**Table S5.** *Ceriodaphnia dubia* 7 days survival and reproduction Al toxicity test results with Colorado river water (TSS = 61 mg/L and pH 7.6) normal feeding. Aluminum was measured as pH 4 extracted Al on 24h old solutions after media renewal. Toxicity endpoints EC_50_ = 2.4 (2.0 – 2.8) mg/L and EC_10_ = 0.9 (0.1 – 1.1) mg/L.

| pH 4 - Al  (mg/L) | Number of juveniles per adult replicate | | | | | | | | | | Average  Juveniles | Live  Adults | pH |
| --- | --- | --- | --- | --- | --- | --- | --- | --- | --- | --- | --- | --- | --- |
|  | 1 | 2 | 3 | 4 | 5 | 6 | 7 | 8 | 9 | 10 |  |  |  |
| 0.035 | 9 | 18 | 9 | 17 | 12 | 11 | 10 | 9 | 13 | 9 | 11.7 | 10 | 8.23 |
| 0.166 | 8 | 18 | 11 | 11 | 16 | 15 | 11 | 7 | 5 | 11 | 11.3 | 10 | 8.22 |
| 0.301 | 9 | 15 | 10 | 11 | 16 | 18 | 9 | 5 | 11 | 10 | 11.4 | 10 | 8.20 |
| 0.626 | 10 | 19 | 14 | 15 | 15 | 15 | 17 | 10 | 13 | 17 | 14.5 | 10 | 8.19 |
| 1.353 | 7 | 11 | 10 | 9 | 12 | 9 | 8 | 8 | 11 | 5 | 9.0 | 10 | 8.19 |
| 3.118 | 5 | 5 | 5 | 3 | 3 | 5 | 6 | 6 | 3 | 1 | 4.2 | 10 | 8.18 |

**Table S6 –**Summary of the *Ceriodaphnia dubia* 7 days survival and reproduction Al toxicity test results in filtered Colorado river water and normal feeding. Test media pH was 7.6. Aluminum was measured as pH 4 extracted Al on 24h old solutions after media renewal. Toxicity endpoints EC_50_ = 2.5 (2.2 – 2.8) mg/L, EC_10_ = 0.6 (0.4 – 1.0) mg/L.

| pH 4 - Al  (mg/L) | Number of juveniles per adult replicate | | | | | | | | | | Average  Juveniles | Live  Adults | pH |
| --- | --- | --- | --- | --- | --- | --- | --- | --- | --- | --- | --- | --- | --- |
|  | 1 | 2 | 3 | 4 | 5 | 6 | 7 | 8 | 9 | 10 |  |  |  |
| 0.018 | 18 | 21 | 25 | 16 | 13 | 17 | 17 | 17 | 16 | 13 | 17.3 | 10 | 8.30 |
| 0.170 | 16 | 17 | 18 | 17 | 21 | 20 | 19 | 16 | 21 | 17 | 18.2 | 10 | 8.29 |
| 0.277 | 16 | 13 | 20 | 18 | 18 | 18 | 16 | 17 | 19 | 20 | 17.5 | 10 | 8.27 |
| 0.711 | 12 | 14 | 18 | 16 | 23 | 15 | 16 | 6 | 23 | 13 | 15.6 | 10 | 8.25 |
| 1.635 | 8 | 15 | 14 | 13 | 12 | 13 | 12 | 18 | 18 | 6 | 12.9 | 10 | 8.24 |
| 3.400 | 4 | 10 | 0 | 4 | 3 | 5 | 4 | 8 | 7 | 2 | 4.7 | 10 | 8.25 |

**Table S7 –**Summary of the *Ceriodaphnia dubia* 7 days survival and reproduction Al toxicity test results with simulated Colorado river water (no TSS) at pH 6.3 (MES 5 mM) with normal feeding. Aluminum was measured as pH 4 extracted Al on freshly prepared test solutions (time = 0 h) and 24h old solutions after media renewal. Toxicity endpoints EC_50_ = 0.4 (0.3 – 0.4) mg/L and EC_10_ = 0.1 (0.0 – 0.2) mg/L.

| pH4-Al  (mg/L) | Number of juveniles per adult replicate | | | | | | | | | | Average  Juveniles | Live  Adults | pH |
| --- | --- | --- | --- | --- | --- | --- | --- | --- | --- | --- | --- | --- | --- |
|  | 1 | 2 | 3 | 4 | 5 | 6 | 7 | 8 | 9 | 10 |  |  |  |
| 0 | 18 | 10 | 16 | 16 | 16 | 14 | 15 | 12 | 12 | 7 | 13.6 | 10 | 6.35 |
| 0.09 | 11 | 13 | 20 | 16 | 11 | 12 | 19 | 4 | 1 | 14 | 12.1 | 10 | 6.35 |
| 0.16 | 8 | 16 | 15 | 14 | 14 | 9 | 10 | 10 | 6 | 6 | 10.8 | 10 | 6.36 |
| 0.28 | 9 | 13 | 13 | 8 | 13 | 11 | 11 | 4 | 0 | 12 | 9.4 | 10 | 6.36 |
| 0.63 | 0 | 2 | 0 | 2 | 0 | 0 | 0 | 2 | 0 | 0 | 0.6 | 10 | 6.36 |
| 1.40 | 0 | 0 | 0 | 0 | 0 | 0 | 0 | 0 | 0 | 0 | 0.0 | 0 | 6.41 |

**Table S8 –**Summary of the *Ceriodaphnia dubia* 7 days survival and reproduction of the aluminum toxicity test results with simulated Colorado river water plus natural sediment (200 mg/L) at pH 6.3 (MES 5 mM) with normal feeding and 24 hours aged test solutions. Aluminum was measured as pH 4 extracted Al on freshly prepared test solutions (time = 0 h) and 24h old solutions after media renewal. Toxicity endpoints EC_50_ = 0.6 (0.5 – 0.8) mg/L, EC_10_ = 0.4 (0.2 – 0.4) mg/L.

| pH4-Al  (mg/L) | Number of juveniles per adult replicate | | | | | | | | | | Average  Juveniles | % Mortality | pH |
| --- | --- | --- | --- | --- | --- | --- | --- | --- | --- | --- | --- | --- | --- |
|  | 1 | 2 | 3 | 4 | 5 | 6 | 7 | 8 | 9 | 10 |  |  |  |
| 0.18 | 5 | 6 | 9 | 6 | 5 | 9 | 8 | 9 | 4 | 4 | 6.5 | 10 | 6.42 |
| 0.26 | 4 | 9 | 9 | 9 | 4 | 7 | 13 | 8 | 0 | 7 | 7.0 | 10 | 6.44 |
| 0.35 | 4 | 6 | 3 | 2 | 6 | 4 | 10 | 14 | 9 | 7 | 6.5 | 0 | 6.44 |
| 0.46 | 3 | 4 | 4 | 6 | 2 | 6 | 6 | 2 | 7 | 6 | 4.6 | 0 | 6.42 |
| 0.78 | 1 | 4 | 1 | 4 | 0 | 0 | 4 | 1 | 3 | 5 | 2.3 | 0 | 6.41 |
| 1.41 | 3 | 1 | 5 | 1 | 0 | 4 | 0 | 0 | 2 | 6 | 2.2 | 10 | 6.39 |

**Table S9 –**Summary of the *Ceriodaphnia dubia* 7 days survival and reproduction of the aluminum toxicity test results with simulated Colorado river water plus kaolin clay (104 mg/L) at pH 6.3 (MES 5 mM) with normal feeding. Aluminum was measured as pH 4 extracted Al on freshly prepared test solutions (time = 0 h) and 24h old solutions after media renewal. Toxicity endpoints EC_50_ = 1.0 (0.9 – 1.1) mg/L and EC_10_ = 0.5 (0.1 – 0.8) mg/L.

| Al  (mg/L) | Number of juveniles per adult replicate | | | | | | | | | | Average  Juveniles | % Mortality | pH |
| --- | --- | --- | --- | --- | --- | --- | --- | --- | --- | --- | --- | --- | --- |
|  | 1 | 2 | 3 | 4 | 5 | 6 | 7 | 8 | 9 | 10 |  |  |  |
| 0.01 | 3 | 5 | 6 | 9 | 11 | 15 | 8 | 10 | 13 | 5 | 8.5 | 0 | 6.38 |
| 0.10 | 13 | 14 | 5 | 9 | 4 | 11 | 6 | 9 | 13 | 12 | 9.6 | 0 | 6.38 |
| 0.19 | 12 | 12 | 7 | 4 | 8 | 9 | 13 | 7 | 9 | 12 | 9.3 | 0 | 6.37 |
| 0.36 | 8 | 10 | 5 | 8 | 15 | 11 | 11 | 11 | 9 | 15 | 10.3 | 0 | 6.37 |
| 0.73 | 7 | 10 | 4 | 8 | 3 | 8 | 6 | 8 | 10 | 9 | 7.3 | 0 | 6.35 |
| 1.46 | 0 | 0 | 0 | 0 | 0 | 2 | 0 | 5 | 0 | 0 | 0.7 | 80 | 6.33 |

**Table S10 (Toxicity data from S8).** Summary of the *Ceriodaphnia dubia* 7 days survival and reproduction of the aluminum toxicity test results with simulated Colorado river water plus natural sediment (200 mg/L) at pH 6.3 (MES 5 mM) with normal feeding and 24 hours aged test solutions. Aluminum was measured as pH 4 extracted Al and dissolved and total Al on freshly prepared test solutions (time = 0 h) and 24h old solutions after media renewal, was reported. Toxicity endpoints EC_50_ = 0.6 (0.5 – 0.8) mg/L, EC_10_ = 0.4 (0.2 – 0.4) mg/L.

| Nominal Al  (mg/L) | Al in TSS  (mg/L) | Total Al  (mg/L) | Diss. Al  (mg/L) | pH4-Al  (mg/L) | Number of juveniles per adult replicate | | | | | | | | | | Average  Juveniles/adult | % Mortality | pH |
| --- | --- | --- | --- | --- | --- | --- | --- | --- | --- | --- | --- | --- | --- | --- | --- | --- | --- |
|  |  |  |  |  | 1 | 2 | 3 | 4 | 5 | 6 | 7 | 8 | 9 | 10 |  |  |  |
| 0.000 | 21.8 | 21.8 | 0.009 | 0.18 | 5 | 6 | 9 | 6 | 5 | 9 | 8 | 9 | 4 | 4 | 6.5 | 10 | 6.42 |
| 0.125 | 21.8 | 21.9 | 0.009 | 0.26 | 4 | 9 | 9 | 9 | 4 | 7 | 13 | 8 | 0 | 7 | 7.0 | 10 | 6.44 |
| 0.250 | 21.8 | 22.1 | 0.010 | 0.35 | 4 | 6 | 3 | 2 | 6 | 4 | 10 | 14 | 9 | 7 | 6.5 | 0 | 6.44 |
| 0.500 | 21.8 | 22.3 | 0.010 | 0.46 | 3 | 4 | 4 | 6 | 2 | 6 | 6 | 2 | 7 | 6 | 4.6 | 0 | 6.42 |
| 1.000 | 21.8 | 22.8 | 0.010 | 0.78 | 1 | 4 | 1 | 4 | 0 | 0 | 4 | 1 | 3 | 5 | 2.3 | 0 | 6.41 |
| 2.000 | 21.8 | 23.8 | 0.010 | 1.41 | 3 | 1 | 5 | 1 | 0 | 4 | 0 | 0 | 2 | 6 | 2.2 | 10 | 6.39 |

**RED: Data used for plotting**

**Table S11 (Toxicity data from S9).** Summary of the *Ceriodaphnia dubia* 7 days survival and reproduction of the aluminum toxicity test results with simulated Colorado river water plus kaolin clay (104 mg/L) at pH 6.3 (MES 5 mM) with normal feeding. pH4-Al: average pH 4 extracted aluminum value of freshly prepared test solutions (time = 0 h) and 24h old solutions after media renewal, was reported. Toxicity endpoints EC_50_ = 1.0 (0.9 – 1.1) mg/L and EC_10_ = 0.5 (0.1 – 0.8) mg/L.

| Nominal Al  (mg/L) | Al in Kaolin  (mg/L) | Total Al  (mg/L) | Diss. Al  (mg/L) | pH 4-Al  (mg/L) | Number of juveniles per adult replicate | | | | | | | | | | Average  Juveniles/adult | % Mortality | pH |
| --- | --- | --- | --- | --- | --- | --- | --- | --- | --- | --- | --- | --- | --- | --- | --- | --- | --- |
|  |  |  |  |  | 1 | 2 | 3 | 4 | 5 | 6 | 7 | 8 | 9 | 10 |  |  |  |
| 0.000 | 22.0 | 22.0 | 0.004 | 0.01 | 3 | 5 | 6 | 9 | 11 | 15 | 8 | 10 | 13 | 5 | 8.5 | 0 | 6.38 |
| 0.125 | 22.0 | 22.1 | 0.007 | 0.10 | 13 | 14 | 5 | 9 | 4 | 11 | 6 | 9 | 13 | 12 | 9.6 | 0 | 6.38 |
| 0.250 | 22.0 | 22.3 | 0.008 | 0.19 | 12 | 12 | 7 | 4 | 8 | 9 | 13 | 7 | 9 | 12 | 9.3 | 0 | 6.37 |
| 0.500 | 22.0 | 22.5 | 0.009 | 0.36 | 8 | 10 | 5 | 8 | 15 | 11 | 11 | 11 | 9 | 15 | 10.3 | 0 | 6.37 |
| 1.000 | 22.0 | 23.0 | 0.010 | 0.73 | 7 | 10 | 4 | 8 | 3 | 8 | 6 | 8 | 10 | 9 | 7.3 | 0 | 6.35 |
| 2.000 | 22.0 | 24.0 | 0.009 | 1.46 | 0 | 0 | 0 | 0 | 0 | 2 | 0 | 5 | 0 | 0 | 0.7 | 80 | 6.33 |

**RED: Data used for plotting**

**Table S12.** Comparison of nominal and pH 4 measured Al values for two toxicity tests

(Tables S8 and S9) performed in the presence of TSS at pH 6.3.

| **Nominal Al**  **(mg/L)** | **pH 4-Al measured in Two Toxicity Tests**  **(mg/L)** | | **Percent of Nominal Al** |
| --- | --- | --- | --- |
| 0.000 | 0.18 | 0.01 | -- |
| 0.125 | 0.26 | 0.10 | 144 |
| 0.250 | 0.35 | 0.19 | 76 |
| 0.500 | 0.46 | 0.36 | 82 |
| 1.000 | 0.78 | 0.73 | 97 |
| 2.000 | 1.41 | 1.46 | 71 |
| Average % of Nominal = 80 | | | |
